# *Bacillus subtilis* PM5 from Camel Milk Boosts Chicken Immunity and Abrogates *Salmonella entertitidis* Infections

**DOI:** 10.3390/microorganisms11071719

**Published:** 2023-06-30

**Authors:** Ashraf Khalifa, Hairul-Islam Mohamed Ibrahim, Abdullah Sheikh

**Affiliations:** 1 Biological Science Department, College of Science, King Faisal University, P.O. Box 400, Al-Ahsa 31982, Saudi Arabia; himohamed@kfu.edu.sa; 2 Botany and Microbiology Department, Faculty of Science, Beni-Suef University, Beni-Suef 62511, Egypt; 3 Molecular Biology Division, Pondicherry Centre for Biological Sciences and Educational Trust, Pondicherry 605004, India; 4 Camel Research Center, King Faisal University, P.O. Box 400, Al-Ahsa 31982, Saudi Arabia; asheikh@kfu.edu.sa

**Keywords:** *Bacillus*, camel milk, chicken, infection, probiotics

## Abstract

With the practice of a successful livestock industry using antibiotics, which has continued for more than five decades, researchers have long been interested in finding alternatives to antibiotics for poultry production. Probiotics can potentially reduce enteric diseases in livestock and enhance their productivity. The aim of this study was to isolate putative probiotics from camel milk and test them against *Salmonella* infection as well as host immune development. Thirteen different isolates were obtained from six different camel milk samples from dairy farms in Saudi Arabia. Three of the six isolates (PM1, PM2, PM3, PM4, PM5, and PM6) that showed Gram-positive characters reacted negatively to catalase and hemolytic assays. PM1, PM5, and PM6 showed significant nonpolar surface properties (>51% hydrophobic) and potent antimicrobial activities against avian pathogens, namely *S. enterica, S. typhi, S. aureus,* and *E. coli*. PM5 exhibited substantial probiotic traits; therefore, further focus was given to it. PM5 was identified as *Bacillus subtilis* OQ913924 by the 16S rRNA sequencing method and showed similarity matrix > 99%. An in vivo chicken model was used to access the health benefits of probiotics. After *salmonella* infection, the mucosal immune response was significantly increased (*p* < 0.01), and none of the challenge protocols caused mortality or clinical symptoms after infection in intestinal contents. *S. enterica* organ infiltration in the spleen, thymus, and small intestine was significantly reduced in the *B. subtilis* PM5-fed chickens. The *S. enterica* load in chicken feces was reduced from CFU 7.2 to 5.2 in oral-fed *B. subtilis* PM5-fed chickens. Probiotic-fed chickens showed buffered intestinal content and positively regulated the level of butyric acid (*p* < 0.05), and intestinal interleukin 1 beta (IL1-β), C-reactive protein (CRP), and interferon gamma (IFN-γ) levels were reduced (*p* < 0.05). In addition, *B. subtilis* PM5 showed significant binding to peritoneal macrophages cells and inhibited *S. enterica* surface adhesion, indicating co-aggregation of *B. subtilis* PM5 in macrophage cells. It could be concluded that supplementation with probiotics can improve the growth performance of broilers and the quality of broiler chickens against enteric pathogens. The introduction of this probiotic into the commercial poultry feed market in the near future may assist in narrowing the gap that now exists between chicken breeding and consumer demand.

## 1. Introduction

Camel milk has garnered a lot of interest across the globe because of its medicinal and nutritional benefits [1]. Camel milk has been tested for its microflora, and the results confirm the existence of high levels of microbial diversity, with probiotic bacteria being among the common isolates [2]. Probiotics are defined as live microorganisms that, when administered in sufficient quantities, can provide health benefits to the host [3]. A wide array of probiotic bacterial strains has been isolated from various ecological niches, including camel milk. Examples of this are *Pediococcus pentosaceus, Enterococcus faecium*, and *Enterococcus durans* from raw camel milk in Morocco [4], *Bacillus subtilis* in Iran [5], and *Lactiplantibacillus plantarum* in Tunisia [6]. A large body of evidence confirmed the significant beneficial impacts of probiotics on humans [7,8], animals [9], and plants [10].

Throughout the world, people rely on poultry as an inexpensive and accessible source of animal protein. In the poultry industry, microbial pathogen infection is a major threat that might compromise food safety. O’Bryan et al. (2022) estimate that 1.35 million people become infected, 26,500 people are hospitalized, and 420 people die per year in the United States due to *Salmonella*-related foodborne diseases [11]. There are about 2500 different serovars of the enteropathogenic bacterial species *S. enterica*, which is fairly prevalent. More than 2500 different strains of *Salmonella enterica* exist, making it one of the most diverse bacterial species. Virulence and antibiotic resistance in *Salmonella* strains contribute to a higher incidence of disease and death. *S. enterica* is a common bacterium that causes diarrhea, dehydration, and growth retardation in chickens. In commercial flocks, *S. enterica* may be transmitted by polluted environments, tainted feeds, and diseased rodents. Furthermore, there are health concerns because *S. enterica* can easily be transmitted to humans after they have consumed contaminated food. Using antibiotics to treat bacterial illnesses in chickens has the potential to harm human and poultry health and the environment. In addition to presenting serious threats to public health and food security, the widespread misuse of antibiotics is a major contributor to the emergence of infections that may be resistant to the antibiotics now in use. This means that antibiotics may no longer be effective in treating infections in chickens, which can lead to increased mortality rates and decreased productivity. Additionally, the use of antibiotics can disrupt the gut microbiome of chickens, which can lead to digestive problems and other health issues. Antibiotic-resistant bacteria are responsible for an estimated $35 billion in lost productivity in the United States and 4.95 million deaths worldwide by 2022 [12]. In addition to preventing gastrointestinal disorders, *lactobacilli* has been shown to increase body weight and egg weight in chicken models [13,14]. In addition, bacterial feed (*lactobacilli* and *S. enteritidis)* protected against *S. enterica* infection when used as a mixture outside the chicken host [15].

*Bacillus* species are well known worldwide and generally recognized as safe (GRAS) bacteria, despite the fact that some of their members are pathogenic [16]. They are widespread, showing up in places including water, plants, soil, food, humans, and animals. There are 105 species with validly published and correct names of Gram-positive, aerobic, and facultative anaerobic rod-shaped cells in the genus *Bacillus*. The website (https://lpsn.dsmz.de/search?word=*Bacillus*, accessed on accessed on 15 April 2023) provides a comprehensive list of *Bacillus* species. Species of *Bacillus* displayed remarkable positive impacts, including enhancing T-cell responses in chickens [17], alleviating colitis [7], and attenuating neurodegenerative symptoms in mouse models [8].

Probiotics have been reported as a viable antibiotic-sparing method for preventing *Salmonella* sp. Infections in chicken and enhancing their protein content [18]. Camel’s milk could be a fantastic source for discovering new probiotic bacterium species because it is an ideal growth medium for many microorganisms. This work was designed to isolate potential novel probiotic bacteria from camel milk and test their ability to reduce the incidence of *Salmonella* infection and enhance general parameters of health in chickens including physical condition.

## 2. Materials and Methods

### 2.1. Sample Collection and Bacterial Isolation

Six milk samples (each 200 mL) were obtained from local lactating healthy camels (*Camelus dromedaries*) from local camel farms in Al-Ahsa, Saudi Arabia. Before sampling, the udder was washed with 50% isopropanol and sterile water and dried with a single-use towel. The first three streams of milk were flushed away. The milk samples were collected in a sterilized 50 mL falcon tube and stored in an icebox. Samples were transported immediately to the laboratory for further analysis. The samples (500 µL) were spread-plated on de Man Rogosa Sharpe (MRS) agar (Himedia, India) after being diluted in sterile saline (0.85% *w*/*v* NaCl). After 24 h, morphologically distinct colonies were selected and re-streaked on the MRS agar plate to obtain pure isolates. The chosen colonies were tested again in a glucose yeast peptone (GYP) broth with a pH of 4.5. Successfully growing isolates were biochemically checked. The string test was used to measure Gram staining, the H_2_O_2_ drop test was used to check catalase activity, and Mueller-Hinton media (MH)-based blood agar was used to measure hemolysis. Only isolates that were Gram-positive, catalase-positive, and non-hemolytic characteristics were chosen for the subsequent probiotic features.

### 2.2. Probiotic In Vitro Characterization of Milk Isolates

The measurement of the capacity of isolates to tolerate challenging gastrointestinal conditions such as low gastric pH and bile salt was carried out in accordance with the method mentioned earlier [18]. After 18 h of incubation at 37 °C, isolates were cultured in MRS broth to achieve the necessary cell number (10^9^ log CFU/mL). In brief, the isolates were plated at the appropriate concentration on a modified GYP broth medium that had either pH 3.0 adjusted or contained 0.3% bile salt (Oxgall). CFU/mL were determined in acid/bile-tolerant inoculation isolates by plating 100 μL on MRS agar.

### 2.3. Cell Surface Hydrophobicity

According to Hairul et al. [19], the hydrophobicity of the isolates was determined by their capacity to adhere to a nonpolar mucosal environment (heptane, Merck Co., Whitehouse Station, NJ, USA). In brief, the isolates of choice were cultured in MRS broth (1 mL), and then heptane (1 mL) was added and vortexed. The polar phase’s optical density was measured at the absorbance (A) of 640 nm using a microplate reader (Biotek, Salem, MA, USA). The numbers show the percentage of hydrophobicity on the cell surface. H% = ([A0 − A]/A0 100), where A is the absorbance of the probiotic suspension after mixing with heptane, and A0 is the absorbance of the probiotic suspension before mixing with heptane. Hydrophobic isolates were defined as those with an adherence value greater than 70%.

### 2.4. Assessment of Antimicrobial Activity on Avian Pathogens

The probiotic characteristics of isolates were assessed for their antibacterial activities against *Salmonella enterica, Salmonella typhimurium, Staphylococcus aureus,* and *Escherichia coli* following the method outlined earlier [19]. Pathogenic isolates were procured from the College of Medicine’s Department of Clinical Microbiology, King Faisal University in Al-Ahsa, Saudi Arabia. Overnight cultures of the potential probiotic isolates were harvested by centrifugation (6000× *g* for 10 min at 4 °C). Exactly, 150 μL of the cell-free lysate (pH 7) (Sigma-Aldrich, Darmstadt, Germany) was added to cell pellets. Standard antibiotic disc (Std) ciproflaxacin (5 µg/disc) was used to compare the probiotic lysates. After incubating the cell-free lysate (150 μL/well) of the screened isolates for 18 h at 37 °C, the diameter of the inhibition zone was measured to determine antimicrobial activity.

### 2.5. Molecular Identification Using 16S rRNA Gene Sequencing

Molecular identification of the probiotic bacterial isolates was carried out using 16S rRNA gene sequences. The chosen isolates’ total DNA was isolated with the use of a DNA purification kit (Qiagen, Madison, WI, USA). The primers used were forward 5′-GAGTTTGATCCTGGCTAG-3′ and the reverse 5′-AGAAAGGAGGTGATCCAGCC-3′, and extracted DNA template (25 ng) was combined with PCR master mix (Ampliqon, Odense, Denmark). Electrophoresis on 1% agarose gel was used to examine PCR results for quality control. Clean-up kit (Millipore, Fisher Scientific, Loughborough, UK) instructions were followed to isolate amplicons of the anticipated size. PCR products were sent to the “Macrogen” for sequencing.

### 2.6. Phylogenetic Analyses

The neighbor-joining approach [20] was used to infer the evolutionary history of the probiotic isolates and other related reference strains. The best tree has a total branch length of 0.20093730. Fourteen different DNA sequences were analyzed. The whole dataset included 1350 unique locations. MEGA7 was used to perform the evolutionary studies [21]. Using the Basic Local Alignment Search Tool (Blast-Bioedit tool), the PM5 16S rRNA gene sequence was analyzed and submitted to the GenBank NCBI database.

### 2.7. Bacterial Strain Preparation

*B. subtilis* PM5 was grown in a broth (modified GYP) with a basal chicken diet for 24 h, which yielded the optimum value of chicken feed. After 18 h of aerobic culture in LB, *S. enterica* was given intraorally using an oral gavage catheter.

### 2.8. Chickens’ Maintenance

The Animal Ethics approval was obtained from the committee of the Deanship of Scientific Research at King Faisal University in Al-Ahsa, Saudi Arabia for all preclinical chicken feed tests (KFU-REC-2022-AUG-Ethics101). In the city of Al-Ahsa, Saudi Arabia, 21 white leghorn chickens were purchased from a commercial hatchery at the age of 7 weeks. The procured chickens were excluded for infectious and vaccination history. Cages made of wire (100 × 80 × 50 cm) were used to house the chickens. The temperature was controlled at 28 ± 2 °C, and the self-light 12 h period was used in the laboratory. The birds were fed a basic meal and given access to water at will (*ad libitum*).

### 2.9. Experimental Design

**Figure 1:**
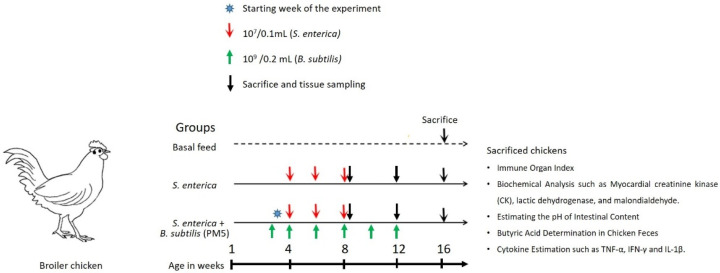
Experimental design to show the effectiveness of *B. subtilis* PM5 as a potential probiotic strain using an in vivo chicken model.

Twenty-one (*n* = 21) chickens were randomly allocated into three experimental groups. Group 1 (*n* = 7, Control) chickens were given a baseline diet. Group 2 (*n* = 7) aimed to assess the effects of orally gavaged sublethal dose *S. enterica* (10^7^ CFU /0.1 mL) on growth, immunological organ index, and inflammatory marker indices. Group 3 (*n* = 7) was studied for the effects of chosen probiotic supplements (*B. subtilis* PM5) (10^9^ CFU /0.2 mL) on *S. enterica*-challenged chickens. A basal meal was provided to seven chickens in the control group (Figure 1).

For four weeks, the treatment group followed a regular diet supplemented with probiotics. The death rates of infected and probiotics-administered chickens were compared after 4 weeks of survival. After 12 weeks of age, the clinical samples were taken for additional investigations including bursal, feces, spleen, and thymus tissues.

### 2.10. Immune Organ Index and Growth Performance

On days 4, 7, 14, and 21, the chickens were weighed individually after an overnight fast, and then the data were used to calculate the average body weights on days 4, 7, 14, and 21. Furthermore, at the age of 4 and 8 weeks, the tissues including the bursal, spleen, and thymus gland were separated and weighed after euthanizing each chicken. Then, the immune organ index (immune organ weight, mg/body weight in grams) was calculated.

### 2.11. Biochemical Analysis

On the 29th day of the trial, the control diet group, a chosen probiotic-supplemented diet, and *S. enterica*-infected chickens were sacrificed. Blood and liver tissue samples were collected. Myocardial creatinine kinase (CK), lactic dehydrogenase (LDH, A020-2-2), and malondialdehyde (MDA, A003-1-2) activities were assessed. In all biochemical tests, total protein was measured and utilized as an internal control. An Invitrogen avian ELISA kit was used to quantify interferon-gamma (IFN-γ) levels in the blood. The feces of chickens were collected and suspended in 0.1 M PBS. The microbial load of *S. enterica* was quantified from the clear supernatant on the MRS agar plate.

### 2.12. Estimating the pH of Intestinal Content

The contents of the probiotic-treated and *Salmonella*-infected chicken gizzard and ileum were aspirated using sterile PBS, and the pH of the contents from the chicken group was determined using a digital pH meter (Mettler Toledo, Greifensee, Switzerland) [22].

### 2.13. Butyric Acid Determination in Chicken Feces

Fecal short-chain fatty acids were identified according to the method previously reported [23] with minor alterations. Briefly, butyrate, the microbial response factor, was measured in fecal samples that were collected, mixed with PBS (four equal volumes), and centrifuged at 12,000× *g* for 15 min. An amount of 1 mM 2-ethylbutyric acid was used as the internal standard. A colorimetric method (Bio-Rad plate reader) was used to quantify the content of butyrate in the fecal samples.

### 2.14. Cytokine Estimation

The cell-free supernatant of chicken serum was used for the estimation of cytokines such as TNF-α, IFN-γ, and IL-1β) [7], TNF-α, IFN-γ, and IL-1β. The concentrations in *S. enterica*-infected and PM5-administered chickens were estimated using enzyme-linked immunosorbent assay (ELISA) kits, and values are expressed as pg/mL (Genway, San Diego, CA, USA).

### 2.15. Assay for Cellular Toxicity and Adhesion

Injecting thioglucolate into mice allowed us to harvest their peritoneal macrophages. Cell counter (Thermo Scientific, Waltham, MA, USA) was used to assess macrophage purity. Dulbecco’s modified Eagle’s medium (DMEM) with 10% fetal calf serum was used to cultivate macrophages. In a 24-well plate, 2105 cells were seeded and incubated for 48 h at 37 °C. After 4 h of induction, cells were infected with *S. enterica* and then washed out with full DMEM. At a multiplicity of infection of 100, *S. enterica*-infected intestinal cells were injected into overnight fresh cultures of PO2 suspended in fresh serum-free DMEM without antibiotics [19]. The survival of macrophage cells, which *S. enterica* invaded via intercellular spaces, was measured under these culture conditions.

### 2.16. Statistical Analysis

The statistical significance of the various treatments was determined using one-way ANOVA on the experimental data at *p* < 0.05. A student’s *t*-test was used to compare the statistical analysis of the probiotic diet group to that of the *Salmonella* group. Microsoft Excel and the SPSS (Statistical Package for the Social Sciences) for Windows (SPSS, Version 10.0, SPSS, Chicago, IL, USA) were used for all statistical analyses.

## 3. Results

### 3.1. Characterization of Camel’s Milk Isolates

Totally, ten different colony morphological isolates were isolated from six camel milk. Only six out of ten isolates reacted positively to Gram staining and negatively to catalase reactions and hemolytic assay (Table 1). These isolates were given the designations PM1, PM2, PM3, PM4, PM5, and PM6 and were chosen for further probiotic characterization.

### 3.2. Impacts of Simulated Bile and Gastric Juice on Isolated Probiotics

**Figure 2:**
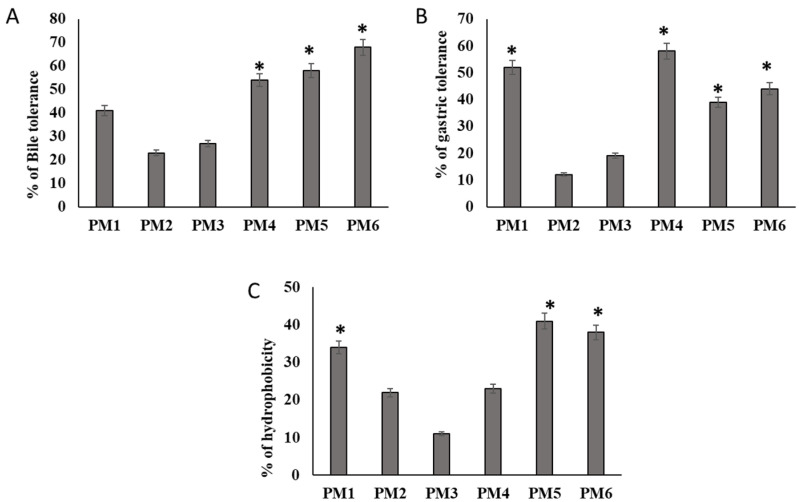
Characteristics of in vitro probiotic activity shown by isolates obtained from camel milk. (**A**) Bile salt 0.6% supplemented in MRS agar medium. Log phase probiotic characteristic isolates (M1, M2, M3, M4, M5, and M6) were inoculated in Oxgall MRS media for 3 h and counted the viable cells as log × CFU. (**B**) The effect of gastric juice tolerance of selected isolates was evaluated in pH 3 in MRS broth. (**C**) Evaluation of several probiotic isolates with regard to the h numbers indicates means ± SD for triplicate observations. Hydrophobic characteristics of their cell surfaces employing the heptane polarity shift technique. Numbers indicate means ± SD for triplicate findings (significant at * *p* ≤ 0.05).

Figure 2A,B show the survival of the isolates in the presence of MRS at 0.45% bile salts and pH 3.0. Four isolates, PM1, PM4, PM5, and PM6 resisted the stress of bile salt, and the count of viable cells was greater than 2.0 CFU/mL after 180 min in the simulated environment. The other two isolates, on the other hand, did not withstand such conditions. Additionally, the Oxgall resistance of the isolates was proven, and they were able to live after 3 h. At pH 3.0, four isolates had considerably higher viability. After 3 h of incubation, PM1, PM4, PM5, and PM6 showed a significant growth increase. The selected four isolates all had viable cell counts that were more than 30% higher than the set baseline levels (Figure 2A,B).

### 3.3. Hydrophobicity of the Cell Surface

The non-polar characteristics of the cell surfaces of considered isolates, as determined by in vitro microbial adherence to heptane droplets, are shown in Figure 2C. The greatest hydrophobic values (>51% hydrophobic nature) were found in PM1, PM5, and PM6 (Figure 2C). The isolates PM1, PM5, and PM6 were evaluated for further clinical antibacterial clearance and in vivo assessment based on these three physiological features.

### 3.4. Assessment of Antimicrobial Activity against Salmonella spp.

**Figure 3:**
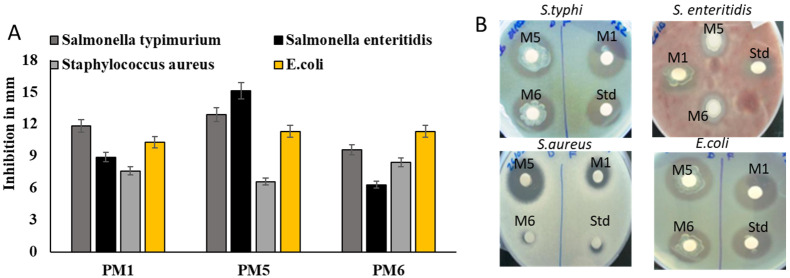
Assessment of selected probiotics on antimicrobial properties against chicken-specific pathogens *E. coli*, *S. aureus, S. enteritidis,* and *S. typhi*. In order to assess the antibacterial activity of isolated bacterial whole lysate against avian infections, plates of MHA were used. (**A**) Antimicrobial activity of selected probiotics against avian pathogens and values expressed in mm in diameter (**B**) MHA plate was used for antimicrobial activity. Std: positive control (Ciprofloxacin 5 µg/disc). The zone of inhibition was calculated in the scale bar and expressed as mm in diameter. Numbers indicate means ± SD for triplicate findings.

The antimicrobial activity of probiotic isolates was tested using avian disease-causing bacteria. The bactericidal action of secretory metabolic products from probiotics was tested against avian pathogens. The isolates PM1, PM5, and PM6 were shown inhibitory activity against *S. typhi*, *S. enterica* and *E. coli*. PM5 showed significant inhibition against all pathogens compared to other tested probiotics. *S. aureus* was not sensitive to probiotic products. The inhibitory zone was substantially larger in PM5, showing that it had robust antibiotic action against *Salmonella* spp. tested. Lysates of PM1, PM5, and PM6 were examined for their anti-*Salmonella* activities to establish if the possible bioactive chemicals that allow antibacterial activity are intracellular or extracellular. PM5 showed significant pathogenic inhibin against both *Salmonella* and *E. coli* strains (Figure 3A,B).

### 3.5. 16S Identification of the Active Isolate

**Figure 4:**
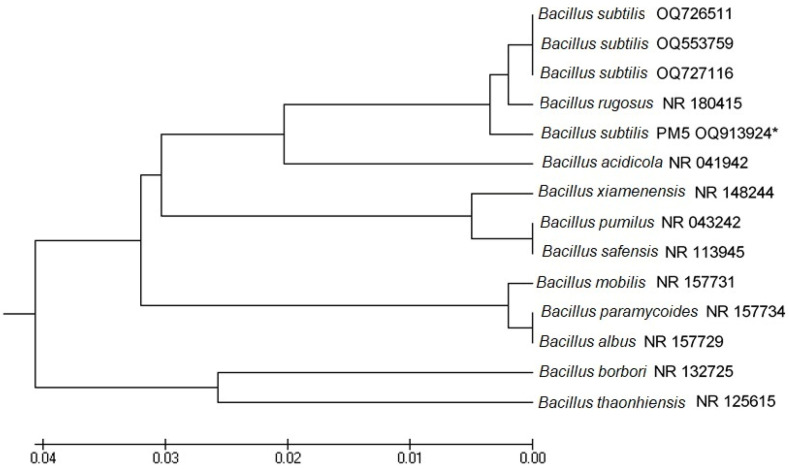
UPGMA similarity relations of the potential probiotic bacterial strains, *Bacillus* subtilis OQ913924 with other closely related strains retrieved from NCBI GenBank. Horizontal bars denote the similarity branch length between the isolates. The scale bar was expressed in 0.01 substitutions per nucleotide position. *: indicates our bacterial isolate used in this study.

The isolates were characterized based on being Gram-positive, catalase-negative rods and then identified based on the 16S rRNA genes. The obtained sequences showed similarity with those of known species available in the NCBI database (Figure 4). 16S rDNA sequences were submitted to GenBank, and the accession number for PM5 (*B. subtilis*) (OQ913924) was 99% similar to that for *B. subtilis* (ON668232). The phylogenetic tree of the neighbor-joining method showed that *B. subtilis* was 0.001 bootstrap closer to *Bacillus* species.

### 3.6. Bacillus Supplementation in the Diet Promotes Chicken Growth Performance

**Figure 5:**
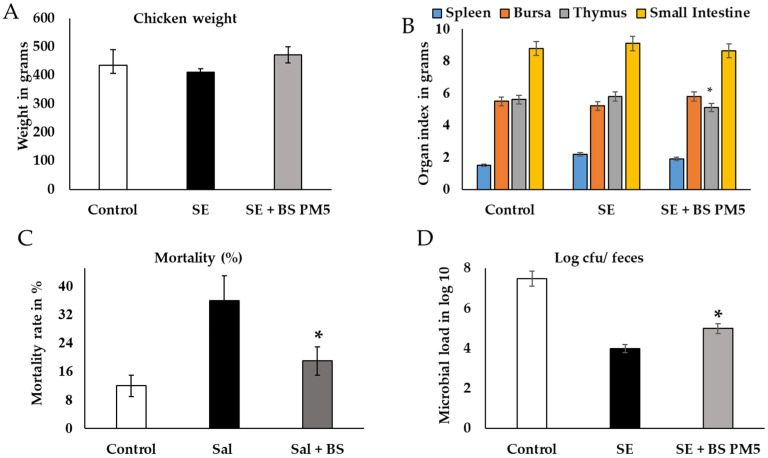
Macroscopic and microbiota analysis of PM5 was evaluated in *Salmonella*-challenged chicken. (**A**) Chicken physiological parameters were evaluated after 28 days of challenge and PM6 administration. The weight of chicken is expressed in grams. (**B**) The organ index indicates the infection rate and recovery level. (**C**) The mortality rate in %. (**D**) Microbial load in fecal samples of chicken and samples were collected and pooled in different intervals, microbial load expressed in log × 10^7^. Data are presented as the average of three independent measurements ± and expressed in respective units. Similar letter group means are insignificantly different (*p* > 0.05), whereas distinct letter group means are significantly different at * *p* ≤ 0.05.

**Figure 6:**
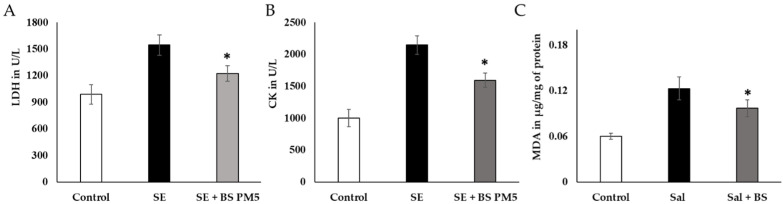
The impact of a PM6-supplemented chicken feed on the immune system’s reaction to *S. enterica* challenge in chickens exposed to extreme strain. (**A**) LDH of liver tissue oxidative stress-related enzymes was evaluated after 4 weeks of acute stress (*n* = 6). (**B**). Serum creatinine kinase levels were analyzed to assess cardiac injury following 4 weeks of PM6 supplementation in chicken (*n* = 6). (**C**) Neutrophil infiltration was measured in hens that were challenged with Salmonella by measuring the activity of malondialdehyde (MDA). In each plot, the values reflect the mean and standard deviation. For repeated measurements, we used one-way ANOVA and then Tukey’s post hoc testing. * shows *p* < 0.05.

Using a chicken model, the probiotic isolate (*B. subtilis* PM5) was tested for in vivo probiotic properties on chicken immune health. The results showed that the effects of *B. subtilis* PM5 on chicken development performance, oxidative stress, and inflammatory cytokines are presented in Figure 5 and Figure 6. When PM5 was supplemented with a chicken feed containing 10^9^ CFU/kg, the mortality of the infected chicken was at a higher rate in *S. enterica* challenged groups than *B. subtilis* PM5-fed chicken (*p* < 0.05). Chickens fed with *B. subtilis* PM5 gained weight, increasing from 427 g to 477 g, and had lower death rates (17 to 34%) (Figure 5). After 4 weeks, significant macroscopic differences in liver tissue color and morphology were observed between *S. enterica*-infected chickens fed with *B. subtilis* PM5 and the control group.

A *B. subtilis* PM5-supplemented diet significantly reduced the infection rate of mass in tissues of the spleen and other organs of immunity (thymus) revealing considerably lower *S. enterica* total count ((*p* < 0.05). Levels of CFU in the bursa and thymus tissues were considerably higher in the chicken group that was only exposed to *S. enterica* (*p* < 0.05). After the treatment period, CFU in the thymus organ elevated substantially (*p* < 0.01) in the 10^9^ CFU/kg *B. subtilis* PM5 group (Figure 5A,B). The small intestine of *S. enterica*-challenged group reduced the CFU of *Salmonella* in a nonsignificant manner. The fecal load of *S. enterica* in orally treated *S. enterica* and *B. subtilis* PM5 was decreased from CFU 6.8 to 3.9/g. Thus, *B. subtilis* PM5-supplemented feed reduced *S. enterica* colonization in the digestive tract significantly (*p* < 0.01) (Figure 5D). Based on the foregoing findings, *B. subtilis* PM5-fed hens were given a meal containing 10^9^ CFU/kg for the following pre-clinical tests.

### 3.7. Bacillus Subtilis Inhibits Oxidative Stress in Chicken

Blood levels of inflammatory and oxidative stress indicators LDH, CK, and MDA were significantly higher in the *S. enterica*-infected group (*p* ≤ 0.05; Figure 6A–C), especially 4 weeks after being exposed to the challenge.

There was a significant decrease in serum LDH and CK levels. (*p* < 0.05) in the *B. subtilis* PM5-fed group compared to the disease group (Figure 6A,B). MDA, a neutrophil infiltration marker, was found to be considerably higher in the liver tissues of the *S. enterica*-infected group. In contrast, with *S. enterica* administration, *B. subtilis* PM5-fed hens displayed increased oxidative stress activities (*p* < 0.05) and lower MDA levels (*p* < 0.05) in the illness group (Figure 6C).

**Figure 7:**
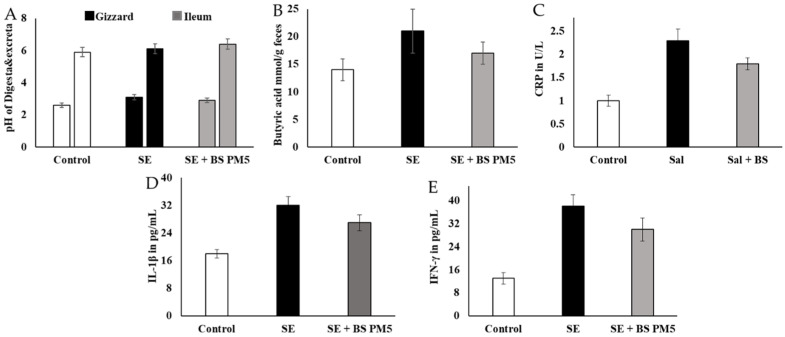
The influence of PM6 supplementation on the inflammatory response of chickens challenged with *S. enterica*. (**A**) pH estimation of different intestinal content (Gizzard and ileum) food content at 20th days of the experiment. (**B**) Estimation of butyric acid levels in the mucus of *S. enterica*-challenged chicken colon and PM5 treatment groups. (**C**) Lymphocyte degradation marker CRP was quantified in the serum samples. (**D**) IL-1β inflammatory cytokine concentration in *S. enterica*-challenged chicken serum and PM5 treatment groups. (**E**) Determination of the IFN-γ content in the serum of *S. enterica*-challenged chickens and PM5-treated groups as an infection stimulation marker cytokine.

The physiological buffer system improves metabolic activity and hormonal regulations. The pH of different parts of food content in *S. enterica*-challenged group and B. subtilis-supplemented group showed significant variation. The gizzard food content was not significant, whereas the ileum food content was significantly increased. The range changes from 5.92 ± 0.2 to 6.13 ± 0.2 The short-chain fatty acids improved the gut–brain metabolic interactions and improve the immune system and hormonal regulation (Figure 7A). Butyric acid levels in *B. subtilis* PM5-fed chickens were higher (*p* < 0.05) than in the control and post-*S. enterica*- infected chicken groups. Butyric acid levels in the *B. subtilis* PM5-fed chicks were significantly increased (*p* ≤ 0.05) (Figure 7B). Probiotic supplementation has a considerable effect on inflammatory stress indicators. In the pro-inflammatory marker comparison research group, *B. subtilis* PM5-fed chickens had considerably lower (*p* < 0.05) intestinal IL-1 and CRP levels, whereas *S. enterica*-infected birds had lower (*p* < 0.05) pro-inflammatory cytokine CRP, IL-1, and IFN- levels (Figure 7C–E).

**Figure 8:**
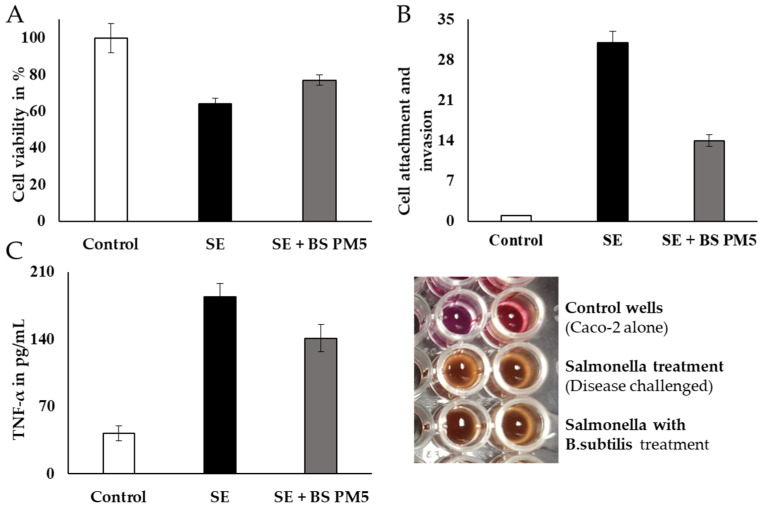
Peritoneal macrophages (PM5) infected with *S. enterica* have been studied for their capacity for survival, invasion, and adhesion. (**A**) The MTT assay was used to measure the viability *of B. subtilis* infected with *Salmonella enterica.* (**B**) The impact of PM6’s adhesion and invasion capabilities on the overall invasion of *S. enterica* in lysed cell content. TNF in *S. enterica*-induced pM5 cells (**C**): PM6’s impact. Mean standard error of the mean (SEM) for triplicate measurements.

The in vitro findings conclude the interaction and co-aggregation of probiotics with pathogens in host immune cells. The cellular model revealed that both the *B. subtilis* PM5 and the non-mutual *S. enterica* displayed considerable binding to peritoneal macrophages in vitro, which was adequate to reduce *S. typhimurium* colonization (Figure 7). The cytotoxicity of peritoneal cell lines was gradually reduced in *B. subtilis* PM5-supplemented group. The peritoneal cell viability was increased from 61% to 72% (Figure 8A). *S. enterica* invasion to peritoneal cells was significantly reduced in the presence of *Bacillus* PM5 (*p* < 0.05). Invasion in co-culture groups including *S. enterica* and B. subtilis PM5 were reported as 29.2 to 13.1 % log CFU/mL, respectively (Figure 8B). The intracellular load of *Salmonella* was determined, and *B. subtilis* PM5-fed cell groups had a substantial decrease in invasive *Salmonella*. These findings showed that *B. subtilis* PM5 showed potent probiotic activity in overcoming infections by *S. enterica* (Figure 8A,B). The septic marker TNF-α was used to assess recovery levels. TNF-α levels in *S. enterica*-infected peritoneal cells were substantially higher than in the infected and control groups. When compared to *Salmonella*-infected cells, *B. subtilis* PM5 strains secrete significantly reduced cytokine levels (Figure 8C).

## 4. Discussion

With the continuous use of antibiotics in the livestock business for more than five decades, researchers have long been interested in developing antibiotic alternatives for poultry production. Probiotics are living microorganisms that are added to animal feed as supplements for boosting health and productivity. In this study, we screened raw camel milk for potential probiotic bacteria strains that could improve chicken resistance against *Salmonella* infections. Out of 13 morphologically distinct isolates, strain PM5 has been selected as a potential probiotic bacterial strain as evidenced by positive Gram staining, negative to catalase and hemolytic tests, the greatest hydrophobic values (>51% hydrophobic nature), and potent antimicrobial activity against avian pathogens, namely *S. enterica*, *S. typhi*, *S. aureus*, and *E. coli*. Furthermore, PM5 displayed a substantial enhancement in chicken health and their resistance against infections with *Salmonella* spp. PM5 has been identified as *B. subtilis* based on the comparative analysis of the 16S rRNA gene sequences.

In order to classify one microbe as a probiotic, a number of attributes should be evaluated at the in vitro and in vivo levels including tolerance to acid and bile salts, hydrophobicity of the cell surface for adhesion, antimicrobial activity, and antibiotic susceptibility [24].

The catalase test for PM5 was negative, indicating that this strain does not generate the catalase enzyme necessary to convert hydrogen peroxide to water and oxygen. This finding also suggests that PM5 can survive on very low levels of oxygen. These results were similar to the study by Saroha et al., who reported Gram-positive, catalase-negative *Limosilactobacillus walteri* sp. as a novel probiotic antimicrobial lipopeptide-producing bacterium [25]. Hemolysis is responsible for the destruction of host cells, and screening bacteria for these products is critical for ensuring the safety of a single isolate [26]. PM5 had a negative hemolytic response, indicating that it is more likely to be a safe isolate with no risk on the host and appropriate for use as probiotics. This finding is consistent with previous reports of *Bacillus subtilis* isolated from camel milk as a probiotic candidate [5].

The acid in the stomach and bile salts in the intestine is the basic bio-barrier that a probiotic strain must pass to reach its target [27]. Low-pH conditions may limit metabolic activity and impair probiotic development and survival. Many studies have shown bacterial viability has been demonstrated to decrease after being exposed to stomach acid at pH 2 for 3 h. [28]. Thus, probiotic strains’ survival and growth in the gastrointestinal tract depend on their tolerance to acid, gastric juice, and bile. In our study, PM5 resisted bile salt, acidic pH, and artificial gastric juice. These results are similar to the probiotic *Bacillus* strain from a previous study [5].

Cell surface hydrophobicity, auto-aggregation, and epithelial cell adherence are other attributes that a probiotic bacterial strain should possess. PM5 showed the highest hydrophobic nature among all other strains. The bacterial film generated by probiotics while sticking to epithelium decreases pathogen–host cell contact [29]. The peritoneal cell line was used to determine specific adherence. In our investigation, the isolate *B. subtilis* PM5 showed strong adhesion and anti-inflammatory action against *S. enterica*. The chosen strain demonstrated colonization with only a few infectious *S. enterica*, adhesion to cells of the epithelium, and development of biofilms according to the hydrophobicity of the surface and auto-aggregation [19,30]. Bacterial adhesion is a complicated mechanism that requires bacterial cell membranes to connect interacting surfaces, giving probiotic bacteria a competitive advantage in undertaking their beneficial action. Although cell surface hydrophobicity has been used as an indicator of in vivo adhesion, a better measure is adhesion assays using cell lines such as Caco2 as a model of the intestinal epithelial barrier. Adhesion experiments that use cell lines such as Caco2 are a better way to measure adhesion in living organisms because they assess the ability of cells to adhere to specific receptors on host cells [31]. The type of cell line (Caco2 or HT29) and bacterial strain are factors that affect the level of bacteria adherence.

PM5 displayed noticeable antimicrobial activities against common avian pathogens, namely *S. typhi, S. enterica* and *E. coli*, as evidenced by the wide zone of inhibition. Comparable results have been obtained with *Bacillus* spp. with probiotic traits [32,33]. Additionally, probiotics have been proven in several in vivo and in vitro investigations to reduce the development of *Salmonella* [9] and *Shigella dysenteriae* [33]. The biocidal action of PM5 can be attributed to the bioactive compounds that the PM5 lysate contained, which negatively affect the pathogen’s metabolic activity, growth, and reproduction. DNA replication, protein synthesis, cell wall integrity, biofilm formation, cell signaling, and membrane integrity are among the potential targets of the bio-compounds [34].

Based on the above-mentioned characteristics, PM5 was confirmed as a potential probiotic bacterial strain obtained from fresh camel’s milk. 16S rDNA sequencing identified PM5 as *B. subtilis* (OQ913924) and was 99.1% similar to that for *B. subtilis*. Furthermore, PM5 was clearly clustered within the *B*. *subtilis* clade based on the phylogenetic tree of 16S rRNA genes from *Bacillus* spp. confirming the identity of the strain. This result also provides further evidence about the effectiveness of the 16S rRNA gene sequencing as a powerful taxonomic tool for bacterial identification at the genus and species levels [35].

The effectiveness of *B. subtilis* PM5 as a potential probiotic strain was further confirmed using an in vivo chicken model. PM5 supplementation in the diet promotes chicken growth performance, boosts the immune response, and enhances resistance against *Salmonella* infections. Similar studies have reported that probiotic strain administration for livestock promotes health and productivity [36]. Peng et al. discovered that a feed enriched with *L. plantarum* increased weight gain in broiler chickens. *B. licheniformis* feeding may enhance body mass and average daily gain (ADG) [37,38]. *S. enterica,* a virulent bacterium, is tightly linked to the chicken gut and causes foodborne diseases that weaken chicken immune systems, resulting in high poultry industry losses [39]. MP5 Supplementation into the diets of chickens infected with *S. enterica* was an effective approach to preventing *S. enterica* from evading the immune system and spreading illness. Genes involved in inflammation and certain cytokines that respond to illness, such as interleukin-8 (IL-8) and interferon-gamma (IFN-γ), are among those whose expression is altered by probiotic bacteria. These findings suggest that improving livestock breeding management is the key to ensuring disease-free livestock. Furthermore, adequate additives for feed may assist in the prevention of bacterial infections such as *E. coli* and *S. enterica* [23,40]. Earlier research has revealed that *L. plantarum* supplementation might enhance intestinal health and reduce mortality in chickens infected with *E. coli* [23]. Furthermore, *Bacillus subtilis*-based probiotic has been found to improve skeletal health and immunity in broiler chickens exposed to heat stress [41] and alleviate neuroinflammation in the hippocampus via the gut microbiota-brain axis in heat-stressed chickens [42]. The recent findings indicate that PM5 has defensive action for combating *S. enterica* by lowering the colonization of *Salmonella*. Butyric acid is produced mainly due to microbial metabolic activities in the intestine and inhibits the growth of pathogenic bacteria [43]. In our investigation, higher amounts of butyric acid and lower detection of *Salmonella* in the ileum were linked to decreased liver cirrhosis in chickens fed with *B. subtilis* PM5. In animal husbandry and poultry research, potential probiotics have emerged as an important, safe, potentially efficient, and sustainable host enhancement approach. These findings showed that *B. subtilis* PM5 could minimize infections caused by bacteria and enhance the quality of broiler chicken. Furthermore, it is possible that the introduction of this probiotic into the commercial poultry feed market in the near future may assist in narrowing the gap that now exists between chicken breeding and consumer demand.

## 5. Conclusions

Herein, we successfully obtained *Bacillus subtilis* strain PM5 from raw camel milk. PM5 was confirmed as a potential probiotic bacterial strain based on positive Gram staining, negative to catalase and hemolytic tests, the highest hydrophobic values, and the potent antimicrobial activity against chicken pathogens, *S. enterica, S. typhi, S. aureus, and E. coli*. Furthermore, PM5 showed a significant improvement in chicken health and resistance to *Salmonella* spp. infections in an in vivo study. These interesting findings open the door for further exploitation of PM5 as an effective and biocompatible antibiotic alternative for sustaining the poultry industry.

## Figures and Tables

**Table 1:** Morphological and physiological characteristics of isolates from camel milk.

Strain Number	Gram Staining	Catalase	Hemolytic	Cell Shape
PM1	Positive	Negative	Negative	Rod
PM2	Positive	Positive	Negative	Cocci chain
PM3	Positive	Negative	Positive	Rod
PM4	Positive	Negative	Negative	Rod
PM5	Positive	Negative	Negative	Rod
PM6	Positive	Negative	Positive	Aggregated rod

## Data Availability

Available from the corresponding author upon request.

## References

[1] Konuspayeva G., Faye B. (2021). Recent Advances in Camel Milk Processing. Animals.

[2] Zhao J., Fan H., Kwok L.-Y., Guo F., Ji R., Ya M., Chen Y. (2020). Analyses of physicochemical properties, bacterial microbiota, and lactic acid bacteria of fresh camel milk collected in Inner Mongolia. J. Dairy Sci..

[3] Hill C., Guarner F., Reid G., Gibson G.R., Merenstein D.J., Pot B., Morelli L., Canani R.B., Flint H.J., Salminen S. (2014). Expert consensus document: The International Scientific Association for Probiotics and Prebiotics consensus statement on the scope and appropriate use of the term probiotic. Nat. Rev. Gastroenterol. Hepatol..

[4] Moussaid S., El Alaoui M.A., Ounine K., Benali A., Bouhlal O., Rkhaila A., Hami H., El Maadoudi E.H. (2023). In-vitro evaluation of the probiotic potential and the fermentation profile of Pediococcus and Enterococcus strains isolated from Moroccan camel milk. Arch. Microbiol..

[5] Daneshazari R., Khorasgani M.R., Hosseini-Abari A., Kim J.-H. (2023). Bacillus subtilis isolates from camel milk as probiotic candidates. Sci. Rep..

[6] Chouikhi A., Ktari N., Bardaa S., Hzami A., Ben Slima S., Trabelsi I., Asehraou A., Ben Salah R. (2021). A novel probiotic strain, Lactiplantibacillus plantarum LC38, isolated from Tunisian camel milk promoting wound healing in Wistar diabetic rats. Arch. Microbiol..

[7] Khalifa A., Sheikh A., Ibrahim H.I.M. (2022). *Bacillus amyloliquefaciens* Enriched Camel Milk Attenuated Colitis Symptoms in Mice Model. Nutrients.

[8] Ibrahim H.I.M., Sheikh A., Khalil H.E., Khalifa A. (2023). *Bacillus amyloliquifaciens*-Supplemented Camel Milk Suppresses Neuroinflammation of Autoimmune Encephalomyelitis in a Mouse Model by Regulating Inflammatory Markers. Nutrients.

[9] Khalifa A., Ibrahim H.I.M. (2023). *Enterococcus faecium* from chicken feces improves chicken immune response and alleviates *Salmonella* infections: A pilot study. J. Anim. Sci..

[10] Al-Tammar F.K., Khalifa A.Y.Z. (2022). Plant growth promoting bacteria drive food security. Braz. J. Biol..

[11] O’Bryan C.A., Ricke S.C., Marcy J.A. (2021). Public health impact of Salmonella spp. on raw poultry: Current concepts and future prospects in the United States. Food Control..

[12] Murray C.J.L., Ikuta K.S., Sharara F., Swetschinski L., Aguilar G.R., Gray A., Han C., Bisignano C., Rao P., Wool E. (2022). Global burden of bacterial antimicrobial resistance in 2019: A systematic analysis. Lancet.

[13] Talebi A., Amirzadeh B., Mokhtari B., Gahri H. (2008). Effects of a multi-strain probiotic (PrimaLac) on performance and antibody responses to Newcastle disease virus and infectious bursal disease virus vaccination in broiler chickens. Avian Pathol..

[14] Taha-Abdelaziz K., Astill J., Kulkarni R.R., Read L.R., Najarian A., Farber J.M., Sharif S. (2019). In vitro assessment of immunomodulatory and anti-Campylobacter activities of probiotic lactobacilli. Sci. Rep..

[15] Juricova H., Matiasovicova J., Faldynova M., Sebkova A., Kubasova T., Prikrylova H., Karasova D., Crhanova M., Havlickova H., Rychlik I. (2022). Probiotic Lactobacilli Do Not Protect Chickens against *Salmonella* Enteritidis Infection by Competitive Exclusion in the Intestinal Tract but in Feed, Outside the Chicken Host. Microorganisms.

[16] Gadotti C. (2011). Control of Pathogenic Bacteria in Queso Fresco by Using Generally Recognized as Safe Ingredients. Ph.D. Thesis.

[17] Larsberg F., Sprechert M., Hesse D., Loh G., Brockmann G.A., Kreuzer-Redmer S. (2023). Probiotic *Bacillus* Strains Enhance T Cell Responses in Chicken. Microorganisms.

[18] El Jeni R., Dittoe D.K., Olson E.G., Lourenco J., Corcionivoschi N., Ricke S.C., Callaway T.R. (2021). Probiotics and potential applications for alternative poultry production systems. Poult. Sci..

[19] Islam V.I.H., Saravanan S., Raj J.P.P., Paulraj M.G., Ignacimuthu S. (2014). Myroides pelagicus from the Gut of Drosophila melanogaster Attenuates Inflammation on Dextran Sodium Sulfate-Induced Colitis. Dig. Dis. Sci..

[20] Saitou N., Nei M. (1987). The neighbor-joining method: A new method for reconstructing phylogenetic trees. Mol. Biol. Evol..

[21] Kumar S., Stecher G., Tamura K. (2016). MEGA7: Molecular Evolutionary Genetics Analysis Version 7.0 for Bigger Datasets. Mol. Biol. Evol..

[22] Sozcu A. (2019). Growth performance, pH value of gizzard, hepatic enzyme activity, immunologic indicators, intestinal histomorphology, and cecal microflora of broilers fed diets supplemented with processed lignocellulose. Poult. Sci..

[23] Ding S., Wang Y., Yan W., Li A., Jiang H., Fang J. (2019). Effects of Lactobacillus plantarum 15-1 and fructooligosaccharides on the response of broilers to pathogenic Escherichia coli O78 challenge. PLoS ONE.

[24] Ayyash M.M., Abdalla A.K., AlKalbani N.S., Baig M.A., Turner M.S., Liu S.Q., Shah N.P. (2021). Invited Review: Characterization of New Probiotics from Dairy and Nondairy Products—Insights into Acid Tolerance, Bile Metabolism and Tolerance, and Adhesion Capability. J. Dairy Sci..

[25] Saroha T., Sharma S., Choksket S., Korpole S., Patil P.B. (2023). *Limosilactobacillus walteri* sp. nov., a novel probiotic antimicrobial lipopeptide-producing bacterium. FEMS Microbiol. Lett..

[26] Haranahalli Nataraj B., Behare P.V., Yadav H., Srivastava A.K. (2023). Emerging Pre-Clinical Safety Assessments for Potential Pro-biotic Strains: A Review. Crit. Rev. Food Sci. Nutr..

[27] Tremblay A., Auger J., Alyousif Z., Calero S.E.C., Mathieu O., Rivero-Mendoza D., Elmaoui A., Dahl W.J., Tompkins T. (2023). Total Transit Time and Probiotic Persistence in Healthy Adults: A Pilot Study. J. Neurogastroenterol. Motil..

[28] Broom L.J., Kogut M.H. (2018). Gut immunity: Its development and reasons and opportunities for modulation in monogastric production animals. Anim. Health Res. Rev..

[29] Noohi N., Papizadeh M., Rohani M., Talebi M., Pourshafie M.R. (2021). Screening for probiotic characters in lactobacilli isolated from chickens revealed the intra-species diversity of *Lactobacillus brevis*. Anim. Nutr..

[30] Mandal A., Mandal R.K., Yang Y., Khatri B., Kong B.-W., Kwon Y.M. (2021). In vitro characterization of chicken gut bacterial isolates for probiotic potentials. Poult. Sci..

[31] Silva-Dias A., Miranda I., Branco J., Monteiro-Soares M., Pina-Vaz C., Rodrigues A.G. (2015). Adhesion, biofilm formation, cell surface hydrophobicity, and antifungal planktonic susceptibility: Relationship among Candida spp. Front. Microbiol..

[32] Simon A., Colom J., Mazhar S., Khokhlova E., Deaton J., Rea K. (2023). *Bacillus megaterium* Renuspore^®^ as a potential probiotic for gut health and detoxification of unwanted dietary contaminants. Front. Microbiol..

[33] Tran C., Horyanto D., Stanley D., Cock I.E., Chen X., Feng Y. (2023). Antimicrobial Properties of *Bacillus* Probiotics as Animal Growth Promoters. Antibiotics.

[34] Moorthy G., Murali M.R., Devaraj S.N. (2007). Protective role of lactobacilli in Shigella dysenteriae 1–induced diarrhea in rats. Nutrition.

[35] Nakharuthai C., Boonanuntanasarn S., Kaewda J., Manassila P. (2023). Isolation of Potential Probiotic *Bacillus* spp. from the Intestine of Nile Tilapia to Construct Recombinant Probiotic Expressing CC Chemokine and Its Effectiveness on Innate Immune Responses in Nile Tilapia. Animals.

[36] Markowiak P., Śliżewska K. (2018). The role of probiotics, prebiotics and synbiotics in animal nutrition. Gut Pathog..

[37] Peng Q., Zeng X.F., Zhu J.L., Wang S., Liu X.T., Hou C.L., Thacker P.A., Qiao S.Y. (2016). Effects of dietary Lactobacillus plantarum B1 on growth performance, intestinal microbiota, and short chain fatty acid profiles in broiler chickens. Poult. Sci..

[38] Chen Y.-C., Yu Y.-H. (2019). Bacillus licheniformis–fermented products improve growth performance and the fecal microbiota community in broilers. Poult. Sci..

[39] Rashid S., Tahir S., Akhtar T., Altaf S., Ashraf R., Qamar W. (2023). Bacillus-Based Probiotics: An Antibiotic Alternative for the Treatment of Salmonellosis in Poultry. Pak. Vet. J..

[40] Forkus B., Ritter S., Vlysidis M., Geldart K., Kaznessis Y.N. (2017). Antimicrobial Probiotics Reduce *Salmonella enterica* in Turkey Gastrointestinal Tracts. Sci. Rep..

[41] Jiang S., Yan F.F., Hu J.Y., Mohammed A., Cheng H.W. (2021). *Bacillus subtilis*-Based Probiotic Improves Skeletal Health and Im-munity in Broiler Chickens Exposed to Heat Stress. Animals.

[42] Fu Y., Hu J., Cheng H.-W. (2023). Research Note: Probiotic, *Bacillus subtilis*, alleviates neuroinflammation in the hippocampus via the gut microbiota-brain axis in heat-stressed chickens. Poult. Sci..

[43] Wang J., Ishfaq M., Guo Y., Chen C., Li J. (2020). Assessment of Probiotic Properties of *Lactobacillus salivarius* Isolated From Chickens as Feed Additives. Front. Veter- Sci..

